# Supplementary material for: Barriers and enablers to the effective implementation of robotic assisted surgery
Source: PLoS One. 2022 Aug 29;17(8):e0273696. doi: 10.1371/journal.pone.0273696 (PMC9423619; doi:10.1371/journal.pone.0273696)
Supplement: S1 File — (DOCX) [file pone.0273696.s001.docx]

**S1 File – Detailed methodological steps involved in the theory-informed implementation action plan**

The following steps detail the processes involved in the development of our theory-informed implementation action plans:

1. Consultation of published guidance on intervention development: The Behaviour Change Wheel (BCW) manual (1) and the Theory and Techniques online tool (2) which details Behaviour Change Techniques (BCTs). The BCW manual specifies evidence-based intervention functions that can be tailored to a specific clinical context, such as Training and Education. The BCTs act as intervention ‘ingredients’: the content of the intervention functions, which detail theory-informed techniques designed to bring about a desired change (in this case, to implement/optimise RAS).
2. Selection of contextually appropriate intervention functions most suited to address salient TDF barriers/facilitators.
3. Selection of appropriate evidence-based BCTs to design the contents of the intervention functions. We selected the BCTs that have been proven to initiate change according to the mechanisms of action specified in the TDF (e.g. the BCT ‘Instruction to perform the behaviour’ has been shown to be effective in targeting the barriers/facilitators that are relevant to the TDF domain ‘Skills’). Where more than one TDF domain influenced a barrier/ facilitator, all evidence-based BCTs relevant to the respective domains were considered. We also considered BCTs most commonly linked to the intervention functions proposed (e.g. Training, Education), as well as other BCTs which may complement intervention content.

**References**

1. Michie S, Atkins L, West R. The behaviour change wheel: A guide to designing interventions. Surrey: Silverback Publishing; 2014.

2. Carey RN, Connell LE, Johnston M, Rothman AJ, de Bruin M, Kelly MP, et al. Behavior Change Techniques and Their Mechanisms of Action: A Synthesis of Links Described in Published Intervention Literature. Annals of Behavioral Medicine. 2018 Oct 10;53(8):693–707.
